# Quantitative Phosphoproteomic Analysis Provides Insights into the Sodium Bicarbonate Responsiveness of *Glycine max*

**DOI:** 10.3390/biom13101520

**Published:** 2023-10-13

**Authors:** Qiang Li, Minglong Li, Huiying Ma, Man Xue, Tong Chen, Xiaodong Ding, Shuzhen Zhang, Jialei Xiao

**Affiliations:** 1 Key Laboratory of Soybean Biology of Chinese Education Ministry, Harbin 150030, China; lstrong@neau.edu.cn (Q.L.);; 2 Key Laboratory of Agricultural Biological Functional Genes, Northeast Agricultural University, Harbin 150030, China

**Keywords:** soybean, phosphoprotein, sodium bicarbonate stress, TMT labeling

## Abstract

Sodium bicarbonate stress caused by NaHCO_3_ is one of the most severe abiotic stresses affecting agricultural production worldwide. However, little attention has been given to the molecular mechanisms underlying plant responses to sodium bicarbonate stress. To understand phosphorylation events in signaling pathways triggered by sodium bicarbonate stress, TMT-labeling-based quantitative phosphoproteomic analyses were performed on soybean leaf and root tissues under 50 mM NaHCO_3_ treatment. In the present study, a total of 7856 phosphopeptides were identified from cultivated soybeans (*Glycine max* L. Merr.), representing 3468 phosphoprotein groups, in which 2427 phosphoprotein groups were newly identified. These phosphoprotein groups contained 6326 unique high-probability phosphosites (UHPs), of which 77.2% were newly identified, increasing the current soybean phosphosite database size by 43.4%. Among the phosphopeptides found in this study, we determined 67 phosphopeptides (representing 63 phosphoprotein groups) from leaf tissue and 554 phosphopeptides (representing 487 phosphoprotein groups) from root tissue that showed significant changes in phosphorylation levels under sodium bicarbonate stress (fold change >1.2 or <0.83, respectively; *p* < 0.05). Localization prediction showed that most phosphoproteins localized in the nucleus for both leaf and root tissues. GO and KEGG enrichment analyses showed quite different enriched functional terms between leaf and root tissues, and more pathways were enriched in the root tissue than in the leaf tissue. Moreover, a total of 53 different protein kinases and 7 protein phosphatases were identified from the differentially expressed phosphoproteins (DEPs). A protein kinase/phosphatase interactor analysis showed that the interacting proteins were mainly involved in/with transporters/membrane trafficking, transcriptional level regulation, protein level regulation, signaling/stress response, and miscellaneous functions. The results presented in this study reveal insights into the function of post-translational modification in plant responses to sodium bicarbonate stress.

## 1. Introduction

Globally, cultivated soybean (*Glycine max* L. Merr.) is one of the most economically important staple crops for human food, animal feed, and industrial products, and provides nearly 70% of the world’s edible proteins, 30% of the world’s dietary vegetable oils, and some nutraceutical compounds such as isoflavones and saponins [1,2]. Moreover, by interacting with symbiotic nitrogen-fixing bacteria, soybean plants can fix nitrogen to produce usable forms of nitrogen for other organisms [3].

However, as a glycophytic species, the productivity and geographical distribution of cultivated soybean are dramatically limited by salt-affected soils which usually cause saline–alkali stress for plants [4,5]. It is estimated that about 830 million hectares of land worldwide is affected by saline–alkali stress, of which the alkalinized soils account for 434 million hectares [6]. In contrast to the saline stress mainly caused by neutral salts such as NaCl and/or Na_2_SO_4_, alkaline salts such as Na_2_CO_3_ and/or NaHCO_3_ are the principal contributors to soil alkalinity. Sodium bicarbonate stress is one of the most severe abiotic stresses affecting plant growth and development. It exerts not only osmotic stress and ion imbalance, but also a high pH and secondary oxidative stresses in plants [7,8]. Hence, increasing plant tolerance to sodium bicarbonate stress is essential, and it is urgent for us to better understand the mechanisms of plant responses to sodium bicarbonate stress.

In recent decades, researchers have found that plants maintain an intracellular ion balance under saline stress mainly through Ca^2+^-dependent protein kinases such as calcium-dependent protein kinases (CDPKs) and CBL-interacting protein kinases (CIPKs) [9]. For instance, the SOS signaling pathway is a well-conserved signaling cascade for Na^+^/K^+^ ion homeostasis in Arabidopsis, in which the core component SOS2 is a CIPK family protein kinase. Similar to SOS2 in the SOS pathway, another CIPK family member PKS5 was reported to play a central role in regulating high pH stress; it phosphorylates and inhibits H^+^-ATPase on the plasma membrane [10]. SnRK2 and the MAPK family kinase-mediated signaling pathways are believed to be essential for osmotic stress tolerance in plants [9,11]. These results were well in line with the idea that the phosphorylation modifications mediated by protein kinases may play critical roles in plant cells perceiving and responding to sodium bicarbonate stress, and a global investigation of phosphoproteins in soybean will not only be important to better understand the roles of some already known protein kinases in response to sodium bicarbonate stress, but also crucial for revealing the precise regulation mechanisms of the soybean signaling networks mediated by phosphorylation modification in response to sodium bicarbonate stress.

With the development of modern phosphoproteomic technologies, a number of studies have been performed to investigate the phosphoprotein profiles in response to various abiotic stresses such as flooding [12,13], high salinity [14,15], drought [16], and aluminum ions [17] in soybean, and thousands of stress-induced DEPs have been identified. However, most of the above studies chose roots as the experimental material, ignoring the distinctive patterns of stress-induced phosphoproteins in different tissues. Moreover, there have been no phosphoproteomic studies in soybeans under sodium bicarbonate stress thus far.

In this study, we employed a combination of Tandem Mass Tag^TM^ (TMT, Thermo Scientific, Waltham, MA, USA) labeling, TiO_2_ affinity chromatography enrichment, and liquid chromatography–tandem mass spectrometry (LC-MS/MS) technologies to investigate the phosphoproteomic changes in both leaf and root tissues of cultivated soybeans. To our knowledge, this is the first phosphoproteomic study in soybeans under sodium bicarbonate stress. We believe that the results obtained from this study not only detail a large number of phosphosites and phosphoproteins in response to sodium bicarbonate stress, but also lay the foundation for uncovering the potential molecular mechanisms of plant sodium bicarbonate stress responses meditated by protein phosphorylation.

## 2. Materials and Methods

### 2.1. Plant Materials, Cultivation, and Stress Treatment

The seeds of cultivated soybeans (*Glycine max* L. Merr. cultivar Hefeng55) were sterilized with a 5% NaClO solution, rinsed in distilled water, and sown in pots filled with a mixture of vermiculite/peat moss/perlite (1:1:1) at 22–25 °C (day 25 °C/night 22 °C, light 16 h/dark 8 h, 1500 ± 200 lx) and at 60% relative humidity, and were germinated by irrigation with water. The seedlings at the V1 stage (about 14 days after sowing) were treated with water (control) or 50 mM NaHCO_3_ solution for 0, 1, 3, 6, or 12 h. The same tissue samples (leaf or root) treated with water or 50 mM NaHCO_3_ solution for different time periods were harvested separately and separated into twelve groups, namely, Leaf_control_1, Leaf_control_2, Leaf_control_3, Leaf_alkali_1, Leaf_alkali_2, Leaf_alkali_3, Root_control_1, Root_control_2, Root_control_3, Root_alkali_1, Root_ alkali_2, and Root_ alkali_3; i.e., each group is composed of samples harvested from the above five time points after sodium bicarbonate treatment, and each experiment was conducted in biological triplicates. The plant materials were immediately frozen in liquid nitrogen and stored at a −80 °C freezer until use.

### 2.2. Protein Extraction, Digestion, TMT Labeling, and TiO_2_ Enrichment

The fresh soybean samples from each biological replicate were ground into a fine powder in liquid nitrogen. The powder samples were used to extract total proteins in SDT lysis buffer (4% SDS, 0.1 M DTT, 100 mM Tris-HCl, pH 7.6) according to Li et al. [18] with minor modifications. The protein concentration was quantified using the bicinchoninic acid (BCA) method [19]. Proteins were digested with trypsin according to the filter aided proteome preparation (FASP) method [20]. After digestion, the resulting peptide solutions for each sample were desalted on a C_18_ cartridge (Millipore). After freeze-drying, the peptides were redissolved for peptide quantification at 280 nm.

One hundred micrograms of peptides from each sample were labeled with different TMT reagents using a TMT reagent kit (Thermo Scientific, Waltham, MA, USA) according to the manufacturer’s instructions. The molecular structure of TMT reagents used in this study and the TMT labeling groups for samples are shown in Figure S1.

Phosphopeptides were enriched using TiO_2_ beads (GL Sciences, Tokyo, Japan). Briefly, after vacuum freeze-drying, the TMT-labeled peptide mixture was re-suspended in 2,5-dihydroxybenzoic acid (DHB) buffer (0.6% DHB, 16% acetonitrile (ACN), 0.02% trifluoroacetic acid (TFA)). Then, TiO_2_ beads were added, and the suspensions were incubated for 40 min with regular agitation. The pellets were packed into plastic tips and centrifuged for 1 min at 5000× *g* to remove the supernatant; then, the beads were, respectively, washed three times with washing buffer 1 (30% ACN, 3% TFA) and washing buffer 2 (80% ACN, 0.3% TFA) to remove non-absorbed material. Finally, the phosphopeptides were eluted using an elution buffer (40% ACN, 15% NH_4_OH). After lyophilization, the phosphopeptides were re-suspended in a 0.1% formic acid (FA) solution and subjected to mass spectral analyses.

### 2.3. LC-MS/MS Analysis

The enriched peptides were injected into an Easy-nLC1000 (Thermo Scientific, USA). The eluted peptides were first separated on an in-house packed C_18_ column (100 μm ID × 20 mm, RP-C18, 5 μm), and then on another in-house packed C_18_ column (75μm ID × 100 mm, RP-C18, 3 μm) with a flow rate of 250 nL/min. The mobile phase buffer with 0.1% FA in water (buffer A) and an eluting buffer with 0.1% FA in ACN (buffer B) were run over a liner 60 min gradient. The Easy-nLC1000 was coupled online to a Q-Exactive HF-X mass spectrometer (Thermo Scientific, USA). The MS data of each sample were acquired at 300–1800 *m/z* at the resolution of 70 k for over 120 min. The top 10 most abundant ions from each MS scan were subsequently dissociated by high-energy collisional dissociation (HCD) in alternating data-dependent mode. The HCD-generated MS/MS spectra were acquired with a resolution no less than 17,500 at *m/z* 200.

### 2.4. Data Processing

The raw files were analyzed in Mascot v2.2 (Matrix Science, London, UK) and Proteome Discoverer v1.4 (Thermo Scientific, USA) and searched against a *Glycine max* peptide database from Uniprot (http://www.uniprot.org/, accessed on 18 November 2021). The database file includes 85,135 nonredundant predicted peptide sequences. The Mascot search parameters included trypsin as a cleavage enzyme; only up to two allowed missed cleavages; carbamidomethyl (C), TMT 10plex (N-term), and TMT 10plex (K) as fixed modifications; oxidation (M), TMT 10plex (Y), Phospho (STY) as variable modifications; 20 ppm as the peptide mass tolerance; 0.1 Da as the fragment mass tolerance; and a 1% false discovery rate (FDR) as the score threshold for peptide identification. The protein ratios were calculated as the median of only unique peptides of the protein, and a phosphosite probability of 0.75 was used as the cut-off for localization of high probability phosphorylation sites.

### 2.5. Bioinformatics Analyses

The significantly enriched phosphorylation motifs were extracted in the MoMo (v5.5.2) program, which incorporates the Motif-X Algorithm [21]. The phosphopeptides were centered at the phosphorylated amino acid and pre-aligned with seven amino acids upstream and downstream of the phosphorylation site. The background dataset was based on soybean proteins and the significance was set at 1 × 10^−6^.

For the prediction of the subcellular localization of the phosphoproteins, an online program, Plant-mSubP (http://bioinfo.usu.edu/Plant-mSubP/, accessed on 16 January 2023) [22], with “PseAACNCCDipep” as the selected prediction module was used. This approach is based on integrated machine learning approaches and can predict with a high accuracy for 11 single localizations and three dual locations of plant cell.

GO and KEGG pathway enrichment analyses were carried out by using the “clusterProfiler” R package. A protein–protein interaction (PPI) analysis was conducted by using the STRING database (http://string-db.org/, accessed on 27 June 2022) and Cytoscape software (v3.9.1) [23].

The prediction and classification of protein kinases were conducted using an online program iTAK (v1.6) (http://itak.feilab.net/cgi-bin/itak/index.cgi, accessed on 3 February 2023) [24].

### 2.6. Yeast Two-Hybrid Assay

Protein–protein interactions were determined by the yeast two-hybrid (Y2H) approach according to Li et al. [25]. Briefly, the full-length CDSs of the candidate genes were cloned into pGBKT7 or pGADT7 vectors, respectively. The vectors of pGADT7-GmSnRK1α and pGBKT7-Gm SnRK1β were used as a positive control. The obtained combinations of bait and prey constructs were co-transformed into Y2H gold yeast competent cells. The transformed yeast cells were grown on selective media for 5–8 days at 28 °C.

### 2.7. Split-LUC Complementation Assay

The full-length CDSs of the candidate genes were cloned into pCAMBIA1300-nLUC or pCAMBIA1300-cLUC vectors, respectively. After transforming into *Agrobacterium tumefaciens* strain GV3101, the obtained bacterial suspensions were co-infiltrated into 4-week-old tobacco leaves. Luciferase (LUC) signals were observed after 48 h of growth.

### 2.8. RNA Extraction, cDNA Synthesis, and Quantitative Real-Time PCR (RT-qPCR)

The total RNAs were extracted from leaf and root tissues of soybean seedlings using an EasyPure Plant RNA kit (Transgen, Beijing, China) according to the manufacturer’s instructions. Reverse transcription of RNA samples was carried out using a Transcript ALL-in-One First-Strand cDNA Synthesis SuperMix kit (Transgen, Beijing, China). Gene-specific primers were designed using an online program: PrimerQuest^TM^ Tool (Integrated DNA Technologies, Shanghai, China) (http://sg.idtdna.com/Primerquest/Home/Index, accessed on 22 October 2022). The SYBR-Green-PCR-Master-Mix system (Toyobo, Osaka, Japan) was used for real-time PCR, and the expression of the soybean glyceraldehyde-3-phosphate dehydrogenase (GAPDH) gene was used as an internal control. Each gene was measured independently in three biological replicates with a minimum of three technical replicates. The relative quantitative analysis of the experimental data was carried out using the 2^–ΔΔCt^ method [26].

## 3. Results and Discussion

### 3.1. Characterization of the Soybean Phosphoproteome under Sodium Bicarbonate Stress

**Figure 1:**
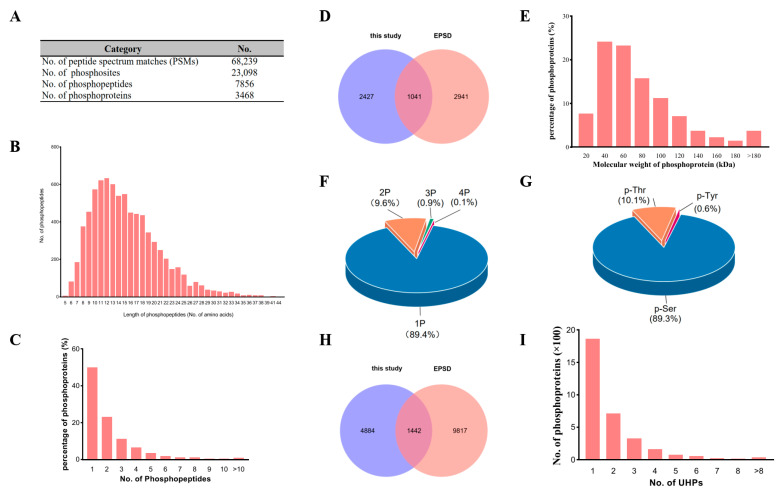
Characteristics of total phosphopeptides and phosphoproteins from *Glycine max* leaf and root samples under sodium bicarbonate stress. (**A**) The statistics of the phosphoproteomic sequencing data; (**B**) the length distribution of the identified phosphopeptides; (**C**) the percentage distribution of phosphoproteins based on the identified phosphopeptides; (**D**) overlap of the phosphoproteins identified in this study and those in the EPSD dataset; (**E**) the percentage of phosphoproteins based on molecular weight; (**F**) the phosphopeptide distribution based on the number of phosphosites (1P, one phosphosites; 2P, two phosphosites; 3P, three phosphosites; 4P, four phosphosites); (**G**) the distribution of phosphorylated serine (p-Ser), phosphorylated threonine (p-Thr), and phosphorylated tyrosine (p-Tyr) sites in the identified phosphopeptides; (**H**) overlap of the UHPs identified in this study and those in the EPSD dataset; (**I**) the frequency of UHPs on phosphoproteins. All phosphoproteomics data are based on biological triplicates.

Our previous study showed that wild soybeans can survive in a nutrient solution with 50 mM NaHCO_3_, and both microarray and RNA-seq analyses identified an inventory of genes with altered expressions regulated by 50 mM NaHCO_3_ stress [27,28,29]. Here, we treated the 14-day-old soybean seedlings with 50 mM NaHCO_3_ for 0, 1, 3, 6, and 12 h and investigated the adverse effects of sodium bicarbonate stress on the growth of soybean. As shown in Figure S2, the soybean seedlings gradually displayed chlorotic symptoms and wilted after sodium bicarbonate stress treatments. Then, we extracted the total proteins from both leaf and root tissues at different time points after sodium bicarbonate stress treatments and fractioned the protein samples on SDS-PAGE gels. The CBB-stained protein electrophoresis patterns were quite different between leaf and root tissues (Figure S3). To understand the underlying mechanisms involved in sodium bicarbonate stress responses in soybean leaf and root tissues, we measured the phosphoproteomic changes in soybean leaf and root tissues under 0 and 50 mM NaHCO_3_. In leaf and root tissues, a total of 68,239 peptide spectrum matches (PSMs) were identified at a 1% false discovery rate (FDR). Of these peptides, 7856 phosphopeptides, assigned to 3468 protein groups (phosphoproteins) with 23,098 non-redundant phosphosites, were detected (Figure 1A, Table S1). The length of non-redundant phosphopeptides varied from 5 to 44 amino acids, and the majority of phosphopeptides (93.7%, 7360/7856) were 7 to 25 amino acids in length (Figure 1B), suggesting that soybean protein digestion by trypsin was successful. These results are comparable with recent phosphoproteomics studies in soybeans [16,17]. More investigations indicated that about half of the phosphoproteins (50.1%, 1739/3468) harbor at least two phosphopeptides (Figure 1C).

In this project, 3468 phosphoproteins were identified. A comparison with the Eukaryotic Phosphorylation Site Database (EPSD) (http://epsd.biocuckoo.cn/index.php, accessed on 6 January 2023) [30] revealed that 1041 phosphoproteins are annotated in the EPSD database, and the other 2427 are unique in this study (Figure 1D), indicating that our study not only greatly enlarges the EPSD phosphoprotein database, but also lays the foundation for future studies on soybean responses to sodium bicarbonate stress. The predicted sizes of the total phosphoproteins vary greatly, ranging from 4.5 to 618.8 kDa (Figure 1E).

In total, 6946 phosphosites with a high probability (≥75%), accounting for 26.9% (6946/23098) of the total non-redundant phosphosites, were identified from 6224 phosphopeptides. Among them, the phosphopeptides with single, double, triple, and quadruple phosphosites accounted for 89.4% (5567/6224), 9.6% (596/6224), 0.9% (57/6224), and 0.1% (4/6224), respectively (Figure 1F). Among all the phosphosites, 6205 (89.3%) are phosphorylated on serine (S) residues, 702 (10.1%) are phosphorylated on threonine (T) residues, and only 39 (0.6%) are phosphorylated on tyrosine (Y) residues (Figure 1G). Although the percentage of phosphorylated Y is very low, phosphoproteomic studies in rice have shown that the Y phosphorylation events in the [TD/EpY] motif are mostly performed by mitogen-activated protein kinases (MAPKs) [31]. In this study, we identified 12 protein kinase domain-containing proteins from a total of 36 phosphoproteins with 39 Y phosphosites, including 6 MAPKs which can phosphorylate the conserved Y residue in the [TD/EpY] motif (Table S2). The GO analysis also showed that the MAPK cascade (GO:0000165) is the most enriched GO term by biological process (BP) classification and the MAP kinase activity (GO:0004707) is the most enriched GO term by molecular function (MF) classification (Figure S4), implying that MAPK signaling may play an important role in the soybean response to sodium bicarbonate stress.

When the high-probability phosphosites were all mapped to the corresponding soybean protein sequences, a total of 6326 unique high-probability phosphosites (UHP) were determined to be located in 3253 phosphoproteins, of which, only 22.8% (1442/6326) UHPs have been identified and recorded in the EPSD database, leaving 77.2% (4884/6326) newly identified UHPs in this study, which expands the size of the EPSD soybean phosphosite database by 43.4% (Figure 1H, Table S3). For the frequency of UHPs on phosphoproteins (Figure 1I), more than half of the phosphoproteins (57.2%, 1860/3253) possess only one UHP, while the largest number of UHPs on a single phosphoprotein (I1LPJ9) was 28, for a nucleolin 1-like protein. To understand the function of the phosphoprotein group with a large number of phosphosites, we defined the phosphoproteins with more than 10 phosphosites as overly phosphorylated phosphoproteins (OPPs). In 3253 UHP-containing phosphoproteins, a total of 20 OPPs were detected (Table S4). A GO analysis shows that OPPs are mostly enriched in regulation of mRNA splicing via spliceosome (GO:0048024) by BP, RNA binding (GO:0003723) by MF, and nuclear lumen (GO:0031981) by cellular components (CCs) (Figure S5). Our findings suggested that OPPs may play key roles in post-transcriptional gene regulation, especially by RNA splicing.

### 3.2. Identification of Differentially Expressed Phosphorylation Events in Response to Sodium Bicarbonate Stress

**Figure 2:**
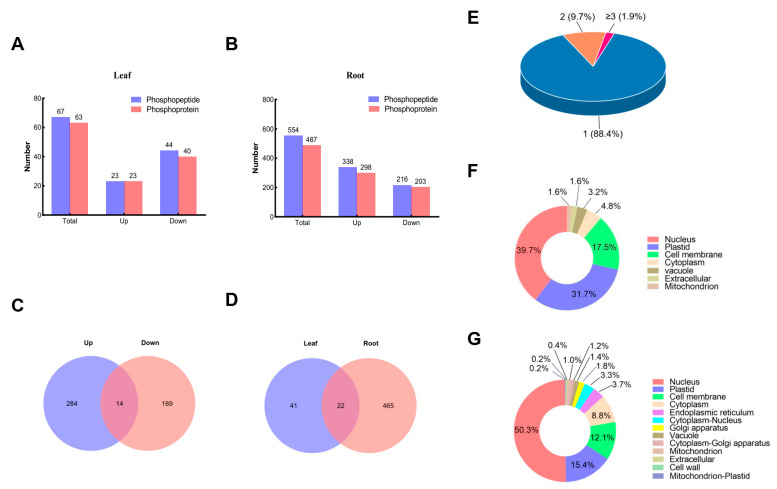
The DEPPs and DEPs from *Glycine max* leaf and root samples under sodium bicarbonate stress. (**A**) Statistics of the sodium bicarbonate stress-responsive DEPPs and DEPs from *Glycine max* leaf samples; (**B**) statistics of the sodium bicarbonate stress-responsive DEPPs and DEPs from *Glycine max* root samples; (**C**) overlap of up-regulated DEPs and down-regulated DEPs identified in *Glycine max* root samples under sodium bicarbonate stress; (**D**) overlap of the DEPs in leaf samples and the DEPs in root samples; (**E**) the percentage distribution of DEPPs in one DEP from *Glycine max* roots and leaves under sodium bicarbonate stress; (**F**) prediction of the subcellular localization of the sodium bicarbonate stress-responsive DEPs from *Glycine max* leaf samples; (**G**) prediction of the subcellular localization of sodium bicarbonate stress-responsive DEPs from *Glycine max* root samples. All phosphoproteomics data are based on biological triplicates.

Based on a *p* value of <0.05 and a threshold of >1.2-fold (ratio of log2) sodium bicarbonate stressed groups vs. the control phosphorylation levels, a total of 67 differential-expressed phosphopeptides (DEPPs), representing 63 DEPs from leaf samples, and a total of 554 DEPPs, representing 487 DEPs from root samples, exhibited significant changes (Figure 2A,B). Among the 63 DEPs from leaf samples, 23 were up-regulated and 40 were down-regulated (Figure 2A and Figure S6A). Similarly, among the 487 DEPs from root samples, 284 were up-regulated, 189 were down-regulated, and another 14 DEPs harboring multi-phosphopeptides appeared in both up- and down-regulated categories (Figure 2B,C and Figure S6B). The number of DEPs obtained from root samples was much higher than those from leaf samples, implying that roots are more sensitive to sodium bicarbonate stress and the response to stress is more active in root tissues.

Notably, 22 DEPs appeared in both leaf and root tissues (Figure 2D), suggesting that common pathways may exist in different plant tissues in the response to sodium bicarbonate stress. Among 528 combined DEPs (represented by 605 total DEPPs) from both tissues, 467 (88.4%) harbor only one unique phosphopeptide, 51 (9.7%) harbor two phosphopeptides, and 10 (1.9%) harbor more than two phosphopeptides. The root-specific up-regulated DEPs (A0A0R0FMI4) harbor up to nine DEPPs (Figure 2E, Tables S5 and S6).

When a more stringent cut-off (fold change (FC) > 2.0 or <0.5, *p* < 0.05) was applied, only 24 DEPPs, harboring 23 UHPs, were identified (Table S7 and Figure S6) from both tissues, representing the most differentially expressed DEPPs in response to sodium bicarbonate stress. Among 24 DEPPs, 13 DEPPs were up-regulated and were all identified from root tissues. The DEPPs with the highest FCs are KEEKVEEESDDDMGLGLFD (FC: 9.88) and KVEEKEESDDDMGFSLFD (FC: 8.37), which are derived from 60S acidic ribosomal proteins. Another 11 DEPPs (root tissue: 9; leaf tissue: 2) were down-regulated. The one with the lowest FC was AGAEASPR (FC: 0.34), derived from an E3 ubiquitin transferase.

Prediction of the protein subcellular localization (Figure 2F,G) shows that, for DEPs from both tissues, most are localized in the nucleus, followed by the plastid, cell membrane, and cytoplasm. However, compared to the DEPs in leaf tissue, more DEPs in root tissues are localized in the nucleus and less are localized in plastids and the cell membrane. Moreover, some DEPs in root tissue were found to localized in two locations in the plant cell (cytoplasm–nucleus, cytoplasm–Golgi apparatus, and mitochondrion–plastid). These results suggest that the roots may play more important roles in the response to sodium bicarbonate stress.

### 3.3. Identification of Phosphor Motifs in the DEPPs

**Figure 3:**
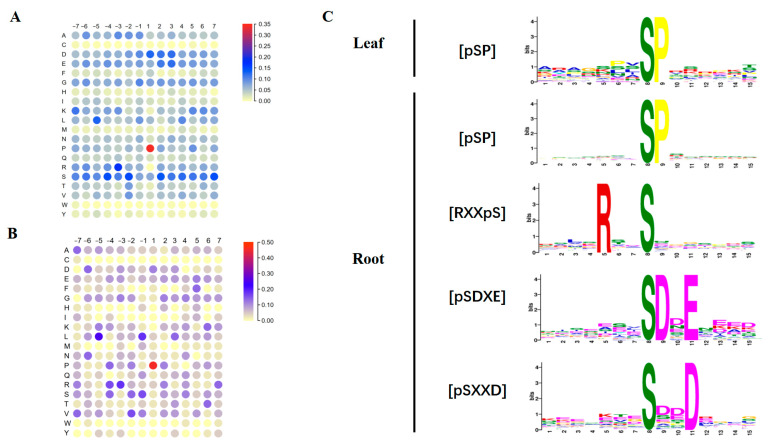
Analyses of the phosphosite flanking sequences. (**A**) The heatmap for the distribution of amino acids in flanking sequences of p-Ser phosphosites. The heatmap was constructed based on 503 unique p-Ser phosphosites; (**B**) the heatmap for the distribution of amino acids in flanking sequences of p-Thr phosphosites. The heatmap was constructed based on 44 unique p-Thr phosphosites; (**C**) the sequence logos of the significantly enriched phosphor motifs extracted from the DEPPs in response to sodium bicarbonate stress in soybean leaf and root tissues. The sequence logos were depicted based on 59 or 460 unique p-Ser phosphosites identified in leaf or root tissues.

In order to reveal the distribution patterns of the amino acids around the phosphosites, the heatmaps for the pre-aligned DEPPs with seven amino acids flanking the phosphosites were visualized. As shown in Figure 3A,B, the amino acid proline (P) at the +1 position is the residue with the highest frequency in the flanking sequences of S and T residues in the DEPPs. This is consistent with the phosphoproteomic results in *Ammopiptanthus mongolicus* roots under short-term drought stress [32], implying some common signaling pathways may exist in plants in the response to both drought and alkaline stresses. In addition, S, glutamate (E), and aspartate (D) also appear at high frequencies in the sequence adjacent to the phosphosite S. In comparison, the amino acid P, leucine (L), and S appear at high frequencies in the sequences adjacent to the phosphosite T.

Different DEPs may be phosphorylated by specific protein kinases. In order to identify these potential protein kinases, the conserved phosphor motifs around the phosphosites were screened. Considering the residue S is a high-probability phosphosite, its flanking sequences in the DEPPs from the *Glycine max* leaves and roots were used to extract the overrepresented phosphor motifs in the Motif-X program. As shown in Table 1 and Figure 3C, [pSP] is the only phosphor motif extracted from DEPPs with pSer in response to sodium bicarbonate stress in soybean leaf tissue, which is also the most abundant phosphor motif extracted from DEPPs in root tissues, with 150 matches. In addition to [pSP], another three phosphor motifs ([RXXpS], [pSDXE], and [pSXXD]) are found in DEPPs in root tissue. The common phosphor motif [pSP] enriched in DEPPs from both leaf and root tissues was reported to be associated with GSK-3, cyclin-dependent kinase (CDK), and MAPK kinases [32], and the [RXXpS] motif only enriched in DEPPs from root tissue may be targeted by SnRK2s and CDPKs [33,34]. In addition, as shown in Figure 3A, the phosphosite S flanked by acidic amino acids E or D may be recognized by casein kinase II [35].

### 3.4. Functional Annotation of DEPs in Sodium-Bicarbonate-Stressed Soybeans

In order to characterize the identified DEPs from leaf or root tissues in response to sodium bicarbonate stress, GO and KEGG enrichment analyses were performed.

**Figure 4:**
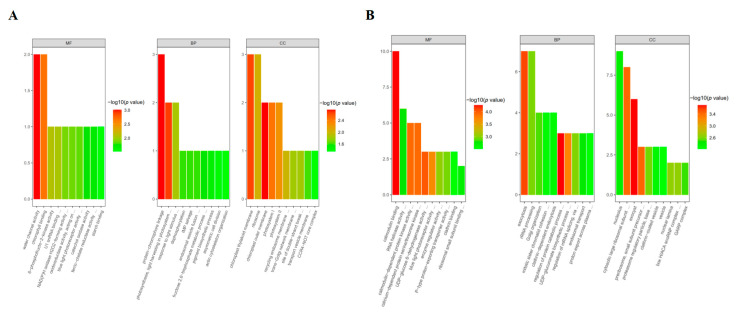
GO analysis of the DEPs from *Glycine max* roots and leaves under sodium bicarbonate stress. (**A**) Leaf samples; (**B**) root samples.

For the GO functional analysis, all identified DEPs were categorized into three functional groups: MF, BP, and CC. As shown in Figure 4 and Tables S8 and S9, the statistically significant GO terms were enriched and were quite different between DEPs from roots and leaves under sodium bicarbonate. In the case of leaf tissue, the overrepresented MF categories include water channel activity (GO:0015250) and chlorophyll binding (GO:0016168); the overrepresented BP categories include protein–chromophore linkage (GO:0018298), photosynthesis, light harvesting in photosystem I (GO:0009768), and response to light stimulus (GO:0009416); and the overrepresented CC categories include chloroplast thylakoid membrane (GO:0009535), ribosome (GO:0005840), chloroplast outer membrane (GO:0009707), and photosystem I (GO:0009522) and II (GO:0009523). However, in the case of root tissue, the MF categories were mainly enriched in calmodulin binding (GO:0005516), RNA helicase activity (GO:0003724), calmodulin-dependent protein kinase activity (GO:0004683), and calcium-dependent protein serine/threonine kinase activity (GO:0009931); the BP categories were mainly enriched in exocytosis (GO:0006887) and rRNA processing (GO:0006364); and the CC categories were mainly enriched in the nucleolus (GO:0005730), cytosolic large ribosomal subunits (GO:0022625), and exocysts (GO:0000145). The differences in the uniquely enriched GO terms between leaf and root tissues may imply that different BPs and MFs are activated in response to sodium bicarbonate stress in different tissues.

**Figure 5:**
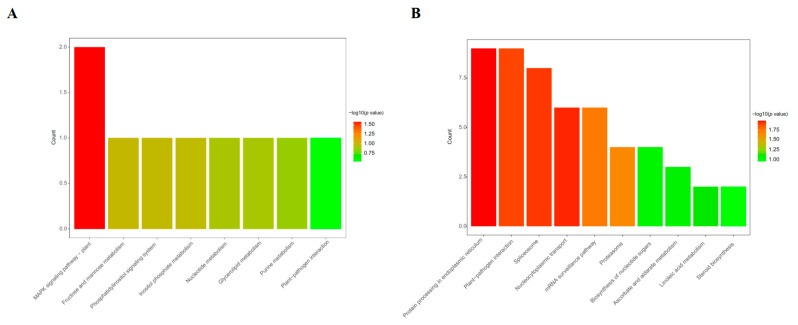
KEGG pathway analysis of the DEPs from *Glycine max* root and leaf samples under sodium bicarbonate stress. (**A**) Leaf samples; (**B**) root samples.

For the KEGG pathway analysis (Figure 5 and Tables S8 and S9), in the case of leaf tissue, the MAPK signaling pathway (gmx04016) is the only statistically significantly enriched (*p*-value < 0.05) KEGG pathway, in which two DEPs (I1KBU7 and I1L345) were involved. However, in the case of root tissue, six statistically significant KEGG pathways were enriched. Nine DEPs (C6T1E8, I1LIQ4, I1K3U6, I1JLS5, C6T7K0, I1KMB9, I1JPP3, A0A0R0KAL0, and I1LEX5) were involved in protein processing in endoplasmic reticulum (gmx04141), nine DEPs (A0A0R0HX26, C6TDV2, P28583, I1JNE0, I1MR60, I1MC08, I1KKF7, K7N5A2, and I1K313) were involved in plant–pathogen interactions (gmx04626), eight DEPs (I1KR32, K7MP06, I1JAI4, I1MRC7, C6T8C8, I1JAG0, I1MYV2, and I1LMC1) were involved in spliceosome (gmx03040), six DEPs (I1JAG0, I1MYV2, I1LQL7, I1LMC1, A0A368UGP3, and I1NGB7) were involved in nucleocytoplasmic transport (gmx03013), six DEPs (I1JAG0, I1MYV2, A0A0R0HUD3, I1L3K1, I1LQL7, and I1LMC1) were involved in the mRNA surveillance pathway (gmx03015), and four DEPs (I1LS70, I1K5H2, C6TGJ3, and I1KQE8) were involved in the proteasome (gmx03050). These results indicate that under sodium bicarbonate stress, the DEPs identified in root tissues could regulate more metabolic pathways than the DEPs in leaf tissues. Moreover, our results reveal the importance of soybean roots in the response to sodium bicarbonate stress, and they may also offer new insights into the different above- and belowground organ-specific responses to abiotic stresses in plants.

### 3.5. Transcriptional Analysis of the DEPs in Response to Sodium Bicarbonate Stress

In order to determine whether the significant changes in the DEP phosphorylation levels result from the alterations in gene expression levels or post-translational modifications, we randomly picked ten DEP-encoding genes for RT-qPCR analysis (Table S10). The products of these DEP-encoding genes include three protein kinases (A0A0R0I4J2, A0A0R4J3S3, and I1JNE0), three splicing factors (I1KR32, I1KIA7, and I1MRC7), one transcription factor (I1M8T1), one E3 ubiquitin ligase (I1MV29), one NADPH-ferrihemoprotein reductase (A0A0R4J338), and one sugar transporter (I1JJW4). Under sodium bicarbonate stress, the phosphorylation levels of all these ten picked DEPs were significantly altered in the root but not leaf tissue. Among them, the phosphorylation levels of four DEPs (I1JNE0, I1KIA7, I1M8T1, and A0A0R4J338) were significantly down-regulated, and the rest were significantly up-regulated. However, in the leaf tissue, as shown in Figure S7A, the expression levels for at least one time point were significantly up-regulated when compared to the 0 h time point in genes encoding A0A0R0I4J2, A0A0R4J3S3, I1MRC7, I1JNE0, and I1JJW4, and were significantly down-regulated in genes encoding A0A0R4J338, I1JNE0, I1KR32, I1M8T1, I1KIA7, and I1JJW4, although no chosen leaf DEP gene was significantly regulated in phosphorylation level. In comparison, in the root tissue, the expression levels at 12 h time point were significantly up-regulated in eight out of ten genes encoding DEPs, including A0A0R4J338, I1KIA7 and I1M8T1 (Figure S7B). Intriguingly, the phosphorylation levels of these three DEPs were down-regulated. Taken together, our results imply that sodium-bicarbonate-stress-induced alterations in gene expression levels are not always correlated with the protein phosphorylation status, and the changes in protein phosphorylation are either due to regulations of the transcriptional level or the post-translational level itself.

### 3.6. Responses of Protein Kinases and Phosphatases to Sodium Bicarbonate Stress

In general, plants adapt to various stresses by activating molecular networks to perform stress perception, signal transduction and expressing specific stress-related genes and metabolites [36]. During these processes, protein kinases and phosphatases (K/Ps) form the backbone of the signaling network in response to various biotic and abiotic stresses by mediating the reversible phosphorylation of proteins.

In this study, a total of 53 different protein-kinase-associated DEPs were identified, accounting for 10.0% (53/528) of the total sodium-bicarbonate-stress-responsive DEPs, in which 48 DEPs containing a kinase domain (PF00069 or PF07714) were determined using the iTAK program, and another 5 DEPs without any obvious protein kinase domain were identified according to annotations in the uniProtKB database (https://www.uniprot.org/, accessed on 18 November 2021). Forty-eight kinase-domain-containing protein kinases belong to six major protein kinase groups: AGC, CAMK, CMGC, RLK, STE, and TKL. Of these protein kinase groups, the receptor-like kinase (RLK) group has the highest proportion (23, 43.4%), followed by the CAMK group (7, 13.2%). These results indicate that post-translational modification by phosphorylation/dephosphorylation plays crucial roles in the plant response to sodium bicarbonate stress.

Numerous studies have shown that the MAPK signaling cascades are involved in plant signal transduction in response to various abiotic stresses [37]. In our study, six MAPK kinases with Tyr phosphosites in the [TD/EpY] motif were identified (Table S2). Moreover, we identified one homologous MAPK (A0A0R0IHB9), two homologous MAP2Ks (I1LBY2 and I1N5P7), and two homologous MAP3Ks (A0A0R0KHG7 and I1L345) from the DEP list (Table S6). It was reported that GmMPK9 (A0A0R0IHB9) functions downstream of ROS to positively regulate guard cell ABA signaling [38]. The MAP3K protein GmYODA (I1L345) is an important negative regulator of stomatal development and has been shown to be regulated by AN3 at the transcriptional level to improve root systems under stress. In addition, the phosphorylation level of YODA was also significantly altered in *Ammopiptanthus mongolicus* roots under drought stress [32,39,40]. In our study, the GmYODA-mediated MAPK cascade is significantly enriched in the KEGG results of DEPs from soybean leaf tissues under sodium bicarbonate stress (Figure 5, Table S8), suggesting the MAPK signaling cascades play a prominent part in the soybean response to sodium bicarbonate stress.

In plants, calcium (Ca^2+^) serves as a conserved second messenger under various environmental stresses, and Ca^2+^-regulating kinases are essential for decoding specific Ca^2+^ signatures elicited by distinct stimuli [41]. In this study, six CDPKs and one CIPK were identified as differentially phosphorylated in soybeans in response to sodium bicarbonate stress, indicating that Ca^2+^-mediated cascades participate in sodium bicarbonate stress signaling in soybeans.

Sucrose-non-fermenting1-related kinase1 (SnRK1) belongs to a conserved hetero-trimeric Ser/Thr kinase family and acts as a key energy and stress sensor. Our previous study revealed the role of SnRK1 as a metabolic regulator under sodium bicarbonate stress [18]. In the present study, the phosphorylation levels of two SnRK1 regulatory subunits (GmSnRK1β (A0A0R0L2D6) and GmSnRK1γ (I1JXJ6)) were up-regulated by sodium bicarbonate stress in the root tissue, further confirming that SnRK1 is linked to sodium bicarbonate stress responsive signaling in soybean roots. Intriguingly, the phosphorylation level of the regulatory subunit of target of rapamycin (TOR) kinase (I1L744) is down-regulated by sodium bicarbonate stress, and it shows interactions with both GmSnRK1β and GmSnRK1γ in our results. Previous studies have pointed out that TOR and SnRK1 interact closely and function in opposite ways in the regulation of nutrient-driven processes (“yin-yang” model) [42]. Our findings prove that the “yin-yang” model is applicable in soybeans under sodium bicarbonate stress.

Additionally, the members of other kinase groups such as AGC, STE, TKL, and RLK/Pelle were also found to have significantly induced phosphorylation levels under sodium bicarbonate stress, but their roles in sodium bicarbonate stress need to be uncovered in the future.

**Figure 6:**
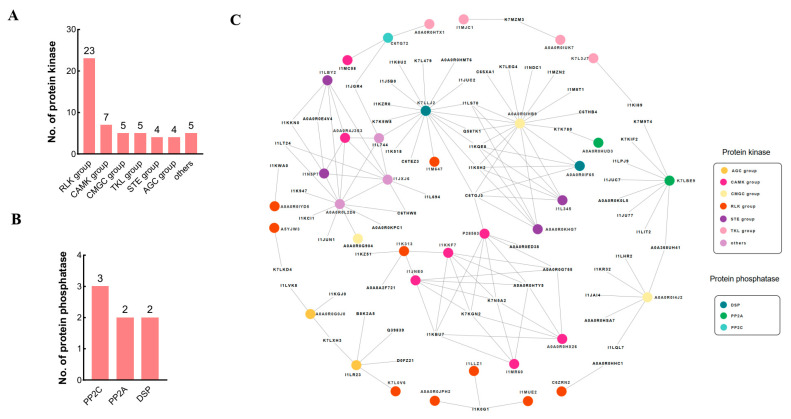
Overview of the protein kinases and phosphatases identified in DEPs from *Glycine max* root and leaf samples under sodium bicarbonate stress. (**A**) The number of protein kinases identified in DEPs from *Glycine max* root and leaf samples under sodium bicarbonate stress; (**B**) the number of protein phosphatases identified in DEPs from *Glycine max* root and leaf samples under sodium bicarbonate stress; (**C**) prediction of the protein kinase/phosphatase-interacting protein network extracted from DEPs of *Glycine max* root and leaf samples under sodium bicarbonate stress.

In addition to protein kinases, seven protein phosphatases, including two regulatory subunits of type 2A protein phosphatases (PP2As) (A0A0R0HUD3 and K7LBE9), three type 2C protein phosphatases (PP2Cs) (C6TG72, I1KMZ8, and I1LT76), and two dual specificity protein phosphatases (DSPs) (GmPHS1 (A0A0R0IF65), GmPTEN2 (K7LLJ2)), were found to be differentially phosphorylated in the soybean response to sodium bicarbonate stress (Figure 6A,B and Table S11). Previous studies have pointed out that PP2A, as the most abundant Ser/Thr protein phosphatase in eukaryotic cells, has multiple functions in plant growth and stress-related signaling, such as ROS signaling, and RBOH, the central player in ROS signaling, is dependent on PP2A at multiple levels. In contrast to PP2A, PP2C is a negative regulator of the ABA signaling pathway, playing important roles in stress signal transduction in plants [43]. In addition, DSP family phosphatase PHS1 is a negative regulator of ABA signaling and is involved in ABA-regulated stomatal opening [44]. PTEN2 in Arabidopsis is an effector of lipid signaling and induced by salt and osmotic stress [45].

Taken together, the K/Ps identified in our results are universally involved in sodium bicarbonate stress signaling in soybeans via reversible phosphorylation and dephosphorylation of their substrate proteins.

### 3.7. The Interactome of Protein Kinases and Phosphatases Responding to Sodium Bicarbonate Stress

To elucidate the functional interactions of the identified protein kinases and phosphatases (K/Ps), a protein–protein interaction (PPI) network containing all differentially phosphorylated K/Ps was constructed. As shown in Figure 6C, a comprehensive interactome representing 101 nodes and 135 edges was obtained. The 101 nodes are composed of 37 K/Ps and 64 interacting protein nodes, and the average edge number per K/P node is 4.3. Further analysis indicates that some K/P nodes have much more connected edges than the average edge number of the total K/P nodes. For example, K7LLJ2 (GmPTEN2, 17 edges), A0A0R0IHB9 (GmMPK9, 16 edges), and A0A0R0L2D6 (GmSnRK1β, 12 edges) are observed to have the highest connectivity, implying these hub nodes may play critical roles in regulating sodium bicarbonate stress responses in soybean. Moreover, according to the annotation results from the UniProtKB database and GO/KEGG analyses, the 64 interacting proteins could be categorized into five classes: membrane trafficking, transcriptional level regulation, protein level regulation, signaling/stress response, and miscellaneous function (Table S12).

Among the 64 interacting proteins, many K/P interactors are annotated as transporters (GmPIN2 (I1LVK8), GmPIP2;5 (A0A0R0ED38), ABC transporters (I1KGJ0 and K7LXH3), GmMSSP2 (A0A0R0G755)) or participate in the process of clathrin-dependent endocytosis (I1JUC2, I1K518, I1K8U2, and K7L479) (Figure 6C). As an auxin efflux transporter, PIN2 has been reported to be involved in the PKS5-mediated signaling cascade under sodium bicarbonate stress by modulating proton secretion in the root tips in Arabidopsis [46]. In the present study, the phosphorylation level of GmPIP2;5, an aquaporin for water transport across the membrane, was significantly changed in both leaf and root tissues, and its ortholog in maize was reported to be a key actor in increasing the sensitivity of stomatal closure to water deficits in a recent study [47]. Thereafter, PIP2;5 may be helpful for mitigating the osmotic stress caused by sodium bicarbonate salts. ABC transporters constitute one of the largest families of membrane proteins and play important roles in the transport of hormones, xenobiotics, metals, and secondary metabolites in plants [48]. In our results, two ABC transporters were identified as the interactors of protein kinases, in which the ortholog of GmABCB19 (K7LXH3) in Arabidopsis was reported to mediate the polar transport of the auxin together with PIN proteins [49]. For another ABC transporter (GmABCG36 (I1KGJ0)), its Arabidopsis ortholog was also thought to be involved in auxin homeostasis in plant cells [50]. In addition, four DEPs (A0A0R0HHC1, A0A0R0E4V4, A0A8A2F721, and I1KKN0) are annotated as proton pump (H^+^-ATPase) interactors (PPIs), suggesting proton pumps might make a great contribution to maintaining the intracellular pH by mediating H^+^ transport across the plasma membrane as well as driving other transporters to alleviate the high pH and ion imbalance caused by sodium bicarbonate salts.

Transcription factors (TFs) are master regulators in controlling various processes of plant development and responses against environmental perturbations. In this study, we detected dozens of TFs from the DEPs of soybeans under sodium bicarbonate stress, of which one bZIP family TF (GmbZIP63 (A0A0R0KPC1)) and two C2H2 family TFs (GmYY1 (C6THB4) and GmZAT10 (I1M8T1)) were identified as the interacting proteins of K/Ps. In Arabidopsis, bZIP63 functions as a key regulator of the starvation response and is a direct target of the SnRK1 kinase [51]. Based on the K/P interactor network, we speculate that GmbZIP63 is phosphorylated by SnRK1 kinase when it interacts with the β subunit of SnRK1 kinase under sodium bicarbonate stress. In addition, YY1 was reported to be a negative regulator of the ABA response network and is involved in salt, drought, and osmotic stresses [52,53]. Moreover, the knockout and RNAi mutants of ZAT10 plants were also more tolerant to osmotic and salinity stress [54]. Our data imply that GmYY1 and GmZAT10 might be phosphorylated and activated by GmMPK9 when exposed to sodium bicarbonate stress.

In addition to the TF-mediated transcriptional regulation, post-transcriptional level regulation via alternative splicing (AS) is also crucial for plants to adapt to environmental stimuli by greatly increasing the transcriptome and proteome complexity. Here, in our study, spliceosome is overrepresented in the KEGG pathway analysis from DEPs of soybean root tissues under sodium bicarbonate stress. Moreover, seven DEPs, including three splicing factors (I1KR32, I1LQL7, and A0A368UH41), one small nuclear ribonucleoprotein (snRNP) GmU1-70K (I1LHR2), one RNA helicase (I1JAI4), and two spliceosomal complex regulatory proteins (A0A0R0HSA7, I1MZN2) were identified as K/P interactors in RNA splicing pathways. GmSC35 (I1KR32) belongs to serine/arginine-rich (SR) protein family, which is a highly conserved family of splicing factors and plays key roles in the execution and regulation of pre-mRNA splicing. Many studies have pointed out that plant SR proteins are phosphoproteins [55]. Our results indicated that GmSC35 interacts with a CMGC group protein kinase GmPRP4KA (A0A0R0I4J2). Intriguingly, GmSC35 is an OPP, with as many as 18 UHPs. Furthermore, U1-70k is a key component of the spliceosome, and the genetic analysis suggested that Arabidopsis U1-70K may be involved in the response to osmotic stress. Taken together, our results suggest that spliceosome-mediated mRNA splicing is regulated by protein phosphorylation under sodium bicarbonate stress.

Under abiotic stress, specific mRNAs are chosen as the templates for new protein synthesis as an adaptive strategy, and these are usually regulated by the reversible phosphorylation of specific eukaryotic translation initiation factors (eIFs) [10,56]. In our data, three eIF-associated DEPs (I1JGR4, I1LIT2, and I1LPJ9) were found to be interacting proteins of K/Ps, and all of these proteins are predicted to participate in eIF4F complex assembly. In eukaryotes, the formation of the eIF4F complex is a rate-limiting step in 5′-cap-dependent translation initiation, becoming a molecular target of translational control [57]. In Arabidopsis, the eIF4F complex is involved in translation regulation under submergence [56]. Our results indicate that the eIF4F complex is also involved in translation regulation under sodium bicarbonate stress. Both biotic and abiotic stress can cause protein misfolding or the accumulation of unfolded proteins. Hence, protein folding is another critical target for protein function regulation. Chaperones are a group of proteins assisting in protein folding in cells under physiological stress conditions. In the present study, one chaperone protein of the heat shock protein (HSP) 70 family (Q587K1), one co-chaperone of HSP90 (C6SXA1), and one HSP70-interacting protein (I1K0G1) were differentially phosphorylated and identified as interactors of K/Ps. These results indicate that the phosphorylation of HSP chaperones is critical for the HSP protein function in the soybean response to sodium bicarbonate stress. In addition, five DEPs (I1K5H2, I1KQE8, I1LS70, K7LKD4, and C6TGJ3) associated with the ubiquitin/26S proteasome degradation system were found to interact with K/Ps under sodium bicarbonate stress. As shown in the KEGG results, the proteasome is significantly enriched in DEPs of soybean root tissue under sodium bicarbonate stress, suggesting the ubiquitin/26S proteasome degradation pathway is regulated by protein phosphorylation under sodium bicarbonate stress. Taken together, our results promote our current understanding of the importance of translational and post-translational regulation via reversible phosphorylation for the plant response sodium bicarbonate stress.

As expected, some signaling- or stress-response-related DEPs were identified as interactors of K/Ps. NADPH oxidase and cryptochrome are the two most important categories. Secondary oxidative stress mediated by reactive oxygen species (ROS) accumulation could be triggered by various abiotic stresses, including sodium bicarbonate stress, causing deleterious effects to membrane lipids, proteins, and nucleic acids in plant cells. A previous study suggested that the respiratory burst oxidase homolog (RBOH) is a calcium-dependent NADPH oxidase and plays a central role in oxidative bursts [58]. In potatoes, RBOHB could be phosphorylated by CDPK5 at Ser82 to regulate oxidative bursts [59]. Here, in this study, we identified four RBOHs (including three RBOHBs and one RBOHC) as interacting proteins of K/Ps in DEPs from soybean root tissue under sodium bicarbonate stress. Interestingly, as shown in the K/P interactor network (Figure 6C), every RBOH identified in this study interacts with five CDPKs (A0A0R0HX26, I1JNE0, I1KKF7, I1MR60, and P28583), implying that CDPKs are redundant in regulating the RBOH function to respond to sodium bicarbonate stress. Cryptochromes (CRYs) are evolutionarily conserved blue-light-absorbing flavoproteins with many important signaling roles in plants. A previous study found that CRY2 in Arabidopsis was involved in blue-light-dependent ROS formation [60], and heterologous expression of wheat CRY1 and CRY2 in Arabidopsis showed a higher sensitivity to high salt levels, osmotic stress, and ABA treatment [61].

To confirm the interactions between K/Ps and their interacting proteins, we examined the physical interactions between six combinations of K/Ps-proteins using Y2H and Split-LUC Complementation assays. For the Y2H assay, all the yeast strains co-transformed with BD-K/Ps and AD-interacting proteins were able to grow normally, as compared to the positive control (Figure S8). For the split-LUC complementation assay, strong LUC signals were detected only in the tobacco leaf regions co-transformed with cLUC-K/Ps and nLUC-interacting proteins (Figure S9). Taken together, our results show bona fide K/P-protein interactions both in yeast and in planta.

**Figure 7:**
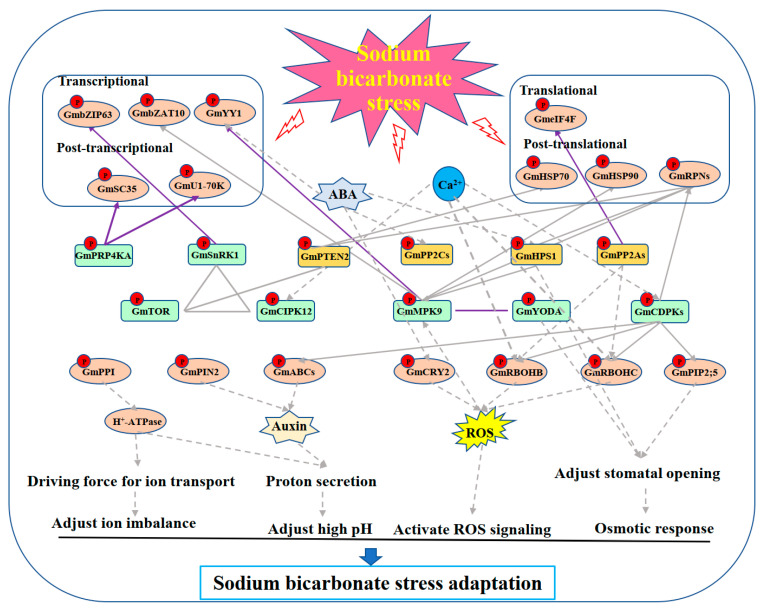
A proposed working model for the response in soybean plants to sodium bicarbonate stress revealed by dynamic phosphoproteomics in the leaf and root tissues. The green rounded rectangle nodes stand for protein kinases. The dark yellow rounded rectangle nodes stand for protein phosphatases. The ellipse nodes stand for interacting proteins of K/Ps. The gray solid lines and arrows indicate the interaction relation between K/Ps and interacting proteins which were revealed in this study. The gray dashed lines and arrows indicate the interactions based on referenced work, and the purple solid lines and arrows indicate the interaction relation between K/Ps and interacting proteins which were verified by Y2H and split-LUC complementation assay in this study.

In summary, according to the results presented in this study and the related references, we propose a working model (Figure 7) for the response of soybean plants to sodium bicarbonate stress revealed by phosphoproteomics in the leaf and root tissues. Sodium bicarbonate stress firstly causes phosphorylation of a series of K/Ps in cells, possibly through Ca^2+^- or ABA-mediated signaling cascades, and then the phosphorylation levels of substrate proteins like transcription factors (GmbZIP63, GmbZAT10, GmYY1), splicing factors (GmSC35, etc.), initiation factors (GmeIF4Fc, etc.), chaperones (GmHSP70 and GmHSP90), and 26S proteasome subunits (GmRPNs) are altered to influence the expression levels of more genes on transcriptional/post-transcriptional and/or translational/post-translational/protein degradation levels. In addition, under sodium bicarbonate stress, the activation of the proton pump (H^+^-ATPase) provides a driving force for ion transport, thereby helping to adjust the ion imbalance caused by sodium bicarbonate stress. Proton efflux is also helpful to reduce the high pH. Similarly, GmPIN2- and GmABC-transporter-mediated auxin efflux also helps to reduce high pH stress. Moreover, sodium bicarbonate stress activates the ROS signaling cascades by phosphorylating GmCRY2 and GmRBOH oxides and alleviates osmotic stress by adjusting stomatal opening. Altogether, the activated pathways lead to soybean adaptation to sodium bicarbonate stress.

## 4. Conclusions

In the present study, we analyzed the global changes in the phosphoproteome in soybean leaf and root tissues under 50 mM NaHCO_3_ treatment using a TMT-labeling-based quantitative phosphoproteomic technology. A total of 7856 phosphopeptides from 3468 phosphoprotein groups in leaf and root tissues were identified. Moreover, a total of 605 DEPPs, representing 528 DEPs in leaf and root tissues under sodium bicarbonate stress, were detected, and from these DEP and DEPPs, the phosphor motifs [pSP], [RXXpS], [pSDXE], and [pSXXD] were extracted. Functional enrichment analyses showed that quite different function terms were enriched between leaf and root tissues, and a larger number of pathways were enriched in the root tissue compared to the leaf tissue. Furthermore, we identified 53 protein kinases and 7 protein phosphatases from the total DEPs and identified 64 interacting proteins of these K/Ps to construct the interactome of K/Ps under sodium bicarbonate stress. Our results reveal insights into the function of phosphorylation/dephosphorylation modification in plant responses to sodium bicarbonate stress.

## Figures and Tables

**Table 1:** Information of the significantly enriched phosphor motifs extracted from DEPPs in response to sodium bicarbonate stress in soybean leaf and root tissues.

Tissue	Motif	Foreground Matches	Foreground Size	Background Matches	Background Size	Fold Enrichment	*p* Value
Leaf	[pSP]	24	56	74,296	1,444,156	8.3	1.0 × 10^−16^
Root	[pSP]	150	444	74,296	1,444,156	6.6	9.8 × 10^−79^
Root	[RXXpS]	81	444	72,669	1,444,156	3.6	1.3 × 10^−23^
Root	[pSDXE]	27	444	5511	1,444,156	15.9	1.4 × 10^−23^
Root	[pSXXD]	53	444	76,745	1,444,156	2.2	4.8 × 10^−8^

## Data Availability

Mass spectrometry proteomics data have been deposited to the ProteomeXchange Consortium via the PRIDE [62] partner repository with the dataset identifier PXD041842. (Username: reviewer_pxd041842@ebi.ac.uk, Password: p7Lu5bPU).

## Supplementary Materials

The following supporting information can be downloaded at: https://www.mdpi.com/article/10.3390/biom13101520/s1, Table S1: Identified phosphopeptides in *Glycine max* under sodium bicarbonate stress; Table S2: Identified tyrosine phosphosites in *Glycine max* under sodium bicarbonate stress; Table S3: Identified UHPs in *Glycine max* under sodium bicarbonate stress; Table S4: Identified OPPs in *Glycine max* under sodium bicarbonate stress; Table S5: Identified DEPs and DEPPs in *Glycine max* under sodium bicarbonate stress; Table S6: The information of DEPs identified in *Glycine max* leaf and root tissues under sodium bicarbonate stress; Table S7: The top differentially expressed DEPPs (FC>2.0) identified in *Glycine max* leaf and root tissues under sodium bicarbonate; Table S8: The results of GO and KEGG analyses of the identified DEPs from leaf tissue under sodium bicarbonate stress; Table S9: The results of GO and KEGG analyses of the identified DEPs from the root tissue under sodium bicarbonate stress; Table S10: Gene specific primer pairs for RT-qPCR; Table S11: Information of K/Ps identified in DEPs from *Glycine max* leaf and root tissues under sodium bicarbonate stress; Table S12: Information of interacting proteins of K/Ps identified in *Glycine max* leaf and root tissues under sodium bicarbonate stress; Figure S1: The TMT labeling groups for protein samples. (**A**) The molecular structure of the six TMT reagents used in this study; (**B**) the TMT labeling groups for all protein samples; Figure S2: Phenotype of 14-day-old soybean seedlings at different time points after sodium bicarbonate stress treatment; Figure S3: SDS-PAGE profile stained with Coomassie brilliant blue (CBB), showing total proteins extracted from *Glycine max* leaf and root tissues at different time points after sodium bicarbonate stress treatment. (**A**) Total proteins extracted from the leaf tissue; (**B**) total proteins extracted from the root tissue. An equal amount (30 μg) of protein was loaded into each well; Figure S4: GO analysis of the phosphoproteins with Y phosphosites identified from *Glycine max* root and leaf samples under sodium bicarbonate stress. (**A**) Biological process; (**B**) molecular function; Figure S5: GO analysis of the overly phosphorylated phosphoproteins (OPPs) identified from *Glycine max* root and leaf samples under sodium bicarbonate stress. (**A**) Biological process; (**B**) molecular function; (**C**) cellular component; Figure S6: Volcano plots of the phosphopeptide distribution in *Glycine max* under sodium bicarbonate stress. (**A**) Leaf tissue; (**B**) root tissue; Figure S7: The RT-qPCR results showing the expression patterns of ten representative DEP-encoding genes under sodium bicarbonate stress treatment. (**A**) The expression patterns in the leaf tissue; (**B**) the expression patterns in the root tissue. GmGAPDH was used as an internal control. Three biological replicates and three technical replicates were used for each experiment. Error bars show the means ± SE of three biological replicates. * and ** indicate significant differences at the 0.05 and 0.01 level by Student’s *t*-tests; Figure S8: Physical interactions between K/Ps and their interacting proteins revealed by a Y2H assay. The K/P-interacting protein combinations are as follows: (**A**) GmSnRK1β (A0A0R0L2D6) and GmbZIP63 (A0A0R0KPC1); (**B**) GmPRP4KA (A0A0R0I4J2) and GmU1-70K (I1LHR2); (**C**) GmPRP4KA (A0A0R0I4J2) and GmSC35 (I1KR32); (**D**) GmMPK9 (A0A0R0IHB9) and GmYODA (I1L345); (**E**) GmMPK9 (A0A0R0IHB9) and GmYY1 (C6THB4); (**F**) GmSIT4 (K7LBE9) and GmNUC-L2 (I1LPJ9); Figure S9: Physical interactions between K/Ps and their interacting proteins revealed by the split-LUC complementation assay in tobacco leaves. The K/P-interacting protein combinations are as follows: (**A**) GmSnRK1β (A0A0R0L2D6) and GmbZIP63 (A0A0R0KPC1); (**B**) GmPRP4KA (A0A0R0I4J2) and GmU1-70K (I1LHR2); (**C**) GmPRP4KA (A0A0R0I4J2) and GmSC35 (I1KR32); (**D**) GmMPK9 (A0A0R0IHB9) and GmYODA (I1L345); (**E**) GmMPK9 (A0A0R0IHB9) and GmYY1 (C6THB4); (**F**) GmSIT4 (K7LBE9) and GmNUC-L2 (I1LPJ9).
