# Supplementary material for: Quantitative Phosphoproteomic Analysis Provides Insights into the Sodium Bicarbonate Responsiveness of Glycine max
Source: Biomolecules. 2023 Oct 13;13(10):1520. doi: 10.3390/biom13101520 (PMC10605096; doi:10.3390/biom13101520)
Supplement: Supplementary file 1 [file biomolecules-13-01520-s001.zip › Supplementary Figures.pdf]

**A**

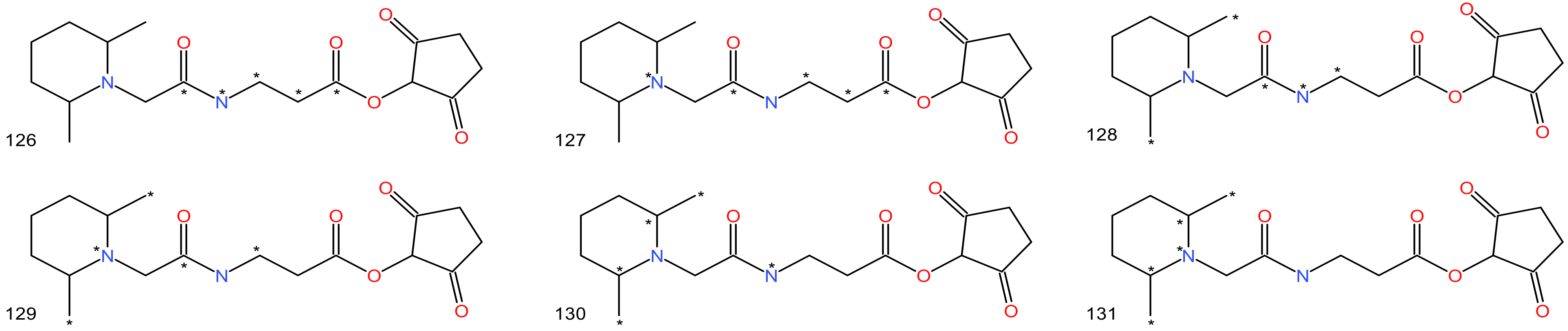


**B**

| Samples | TMT reagent | Group | Samples | TMT reagent | Group |
|---|---|---|---|---|---|
| Leaf_control_1 | 126 | A | Root_control_1 | 126 | B |
| Leaf_control_2 | 127 | A | Root_control_2 | 127 | B |
| Leaf_control_3 | 128 | A | Root_control_3 | 128 | B |
| Leaf_alkali_1 | 129 | A | Root_alkali_1 | 129 | B |
| Leaf_alkali_2 | 130 | A | Root_alkali_2 | 130 | B |
| Leaf_alkali_3 | 131 | A | Root_alkali_3 | 131 | B |

**Figure S1**. The TMT labeling groups for protein samples. (**A**) The molecular structure of six TMT reagents used in this study; (**B**) The TMT labeling groups for all protein samples

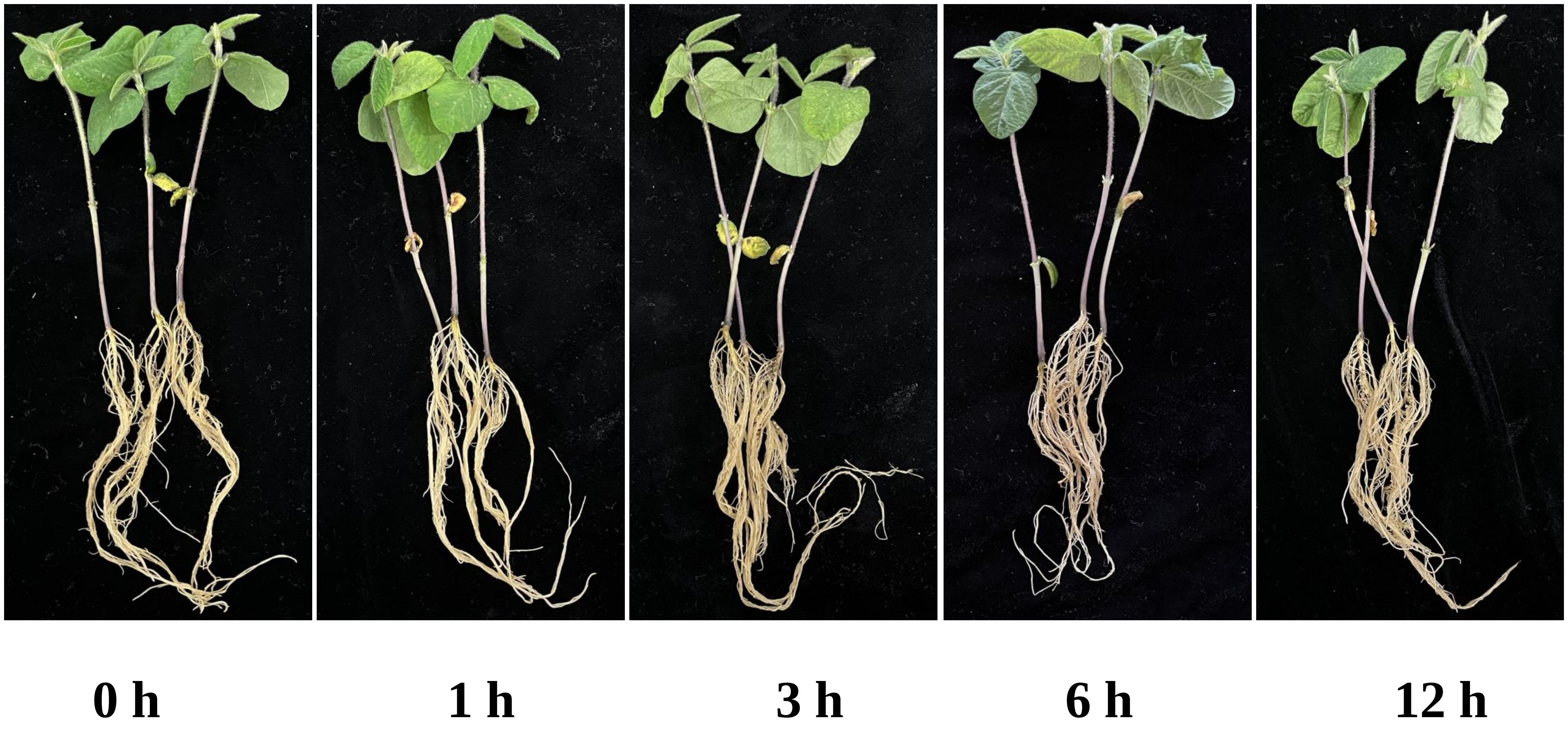


**Figure S2**. Phenotype of 14-day-old soybean seedlings at different time points after sodium bicarbonate stress treatment

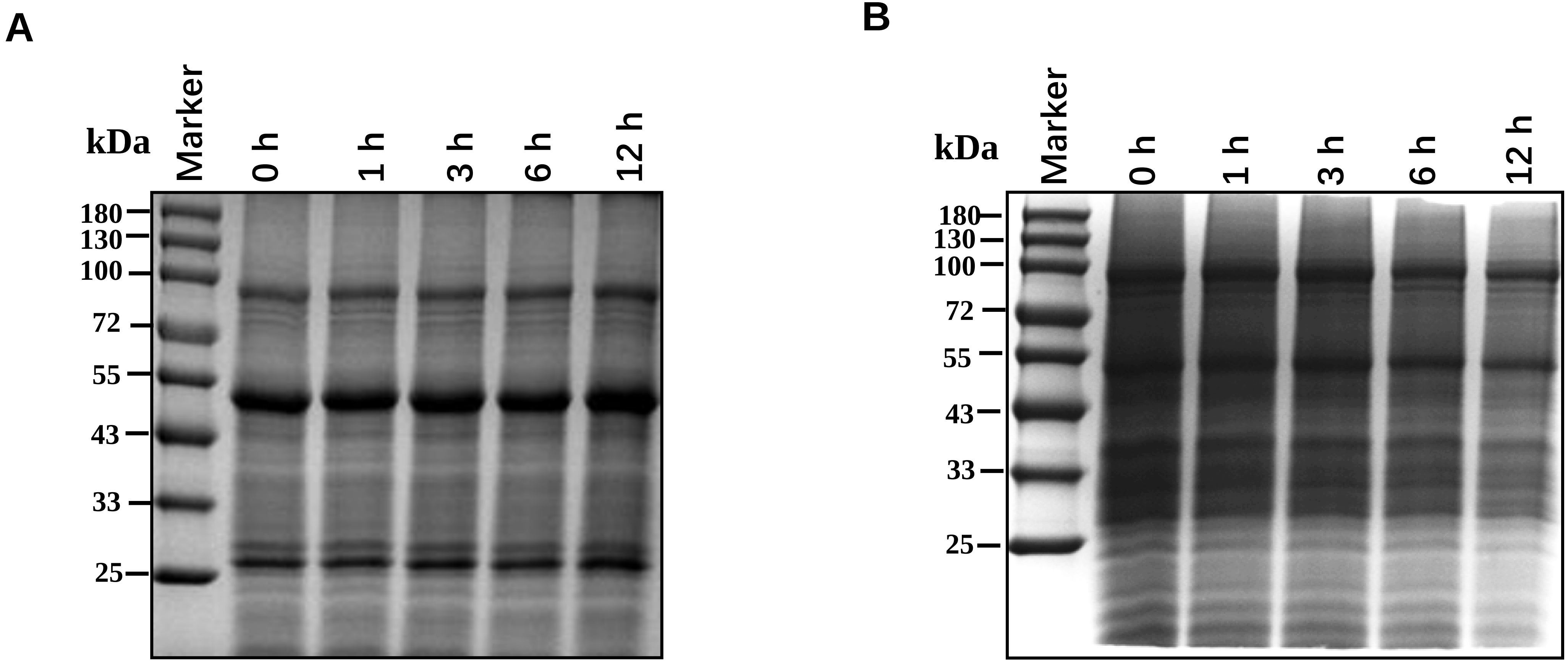


**Figure S3**. SDS-PAGE profile stained with coomassie brilliant blue (CBB) showing total proteins extracted from *Glycine max* leaf and root tissues at different time points after sodium bicarbonate stress treatment. (**A**) Total proteins extracted from the leaf tissue; (**B**) Total proteins extracted from the root tissue. The equal amount (30 μg) of protein was loaded in each well

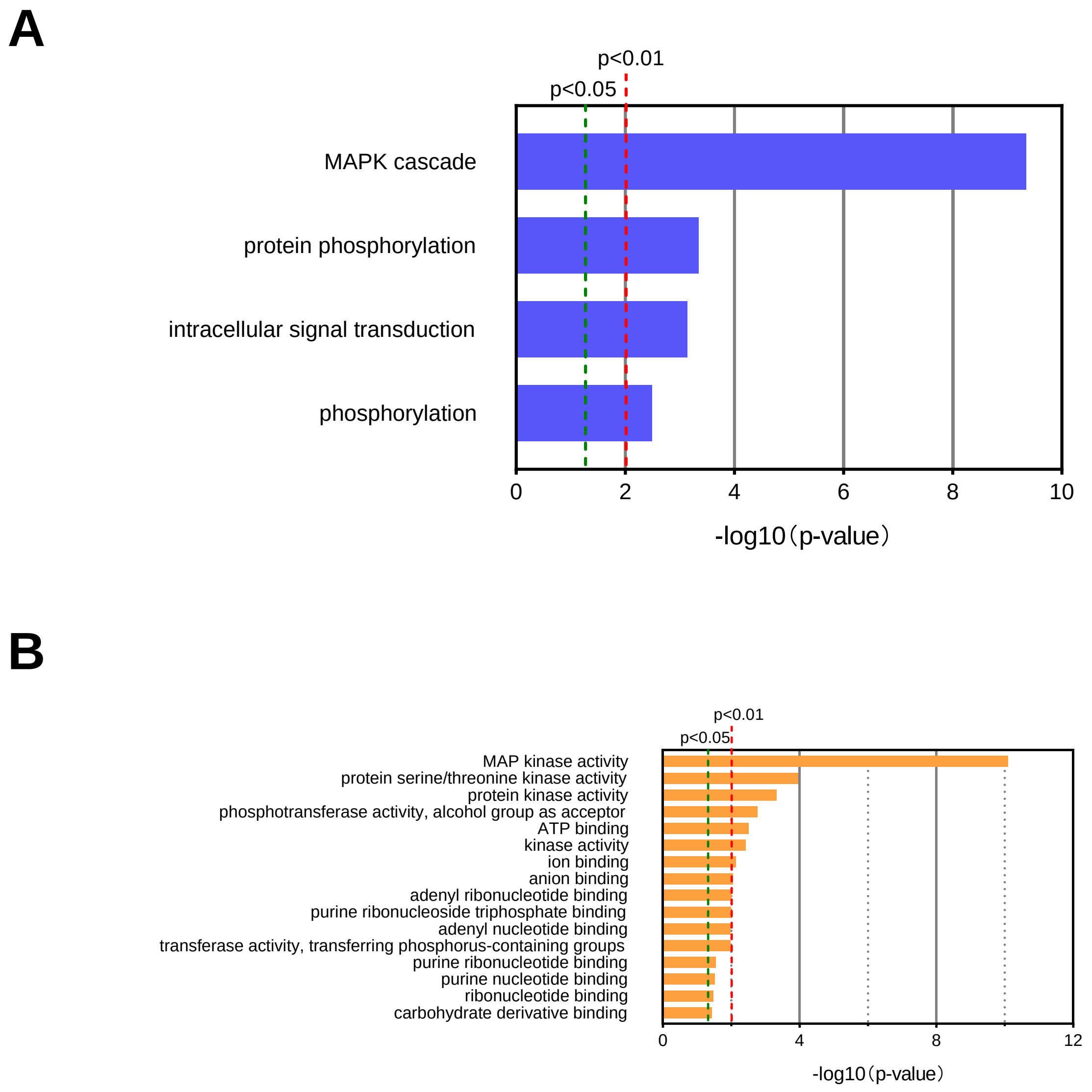


**Figure S4**. GO analysis of the phosphoproteins with Y phosphosites identified from *Glycine max* root and leaf samples under sodium bicarbonate stress. (**A**) Biological Process; (**B**) Molecular Function

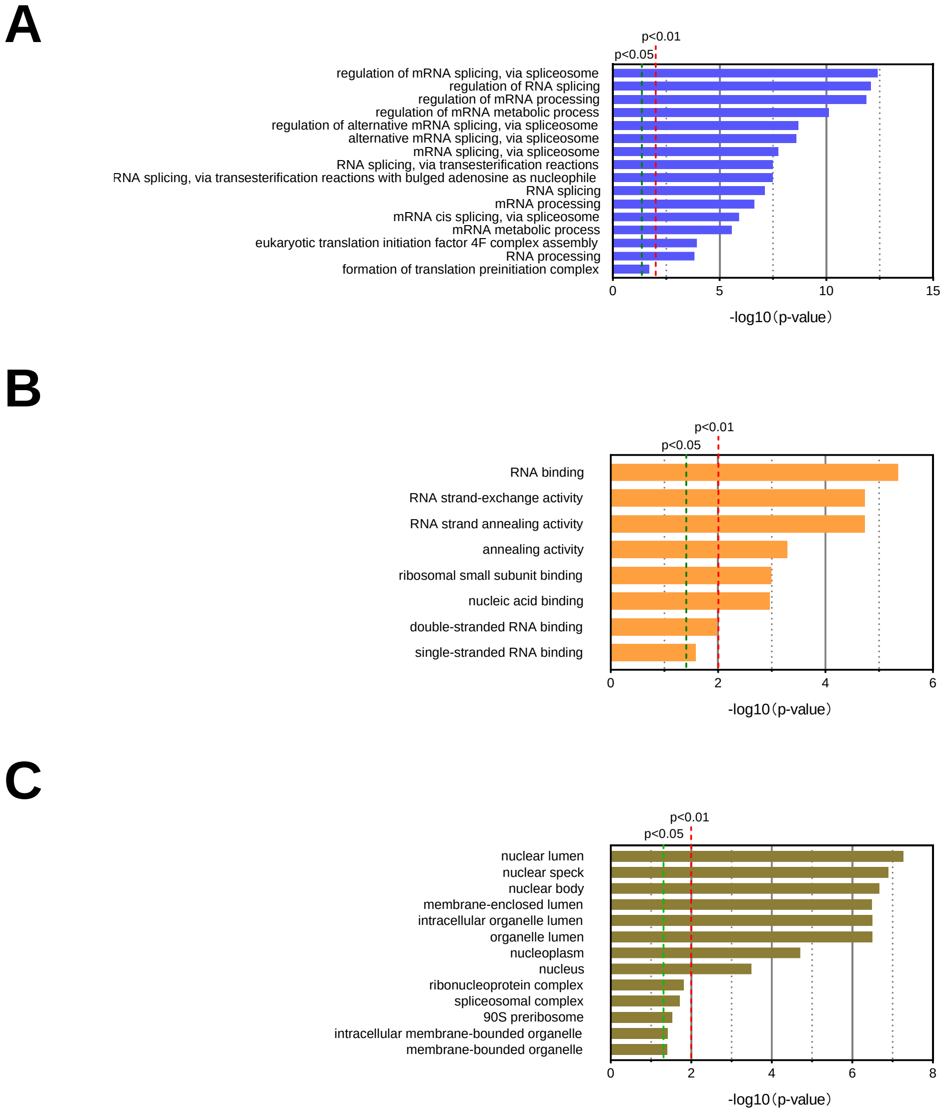


**Figure S5**. GO analysis of the overly phosphorylated phosphoproteins (OPPs) identified from *Glycine max* root and leaf samples under sodium bicarbonate stress. (**A**) Biological Process; (**B**) Molecular Function; (**C**) Cellular Component

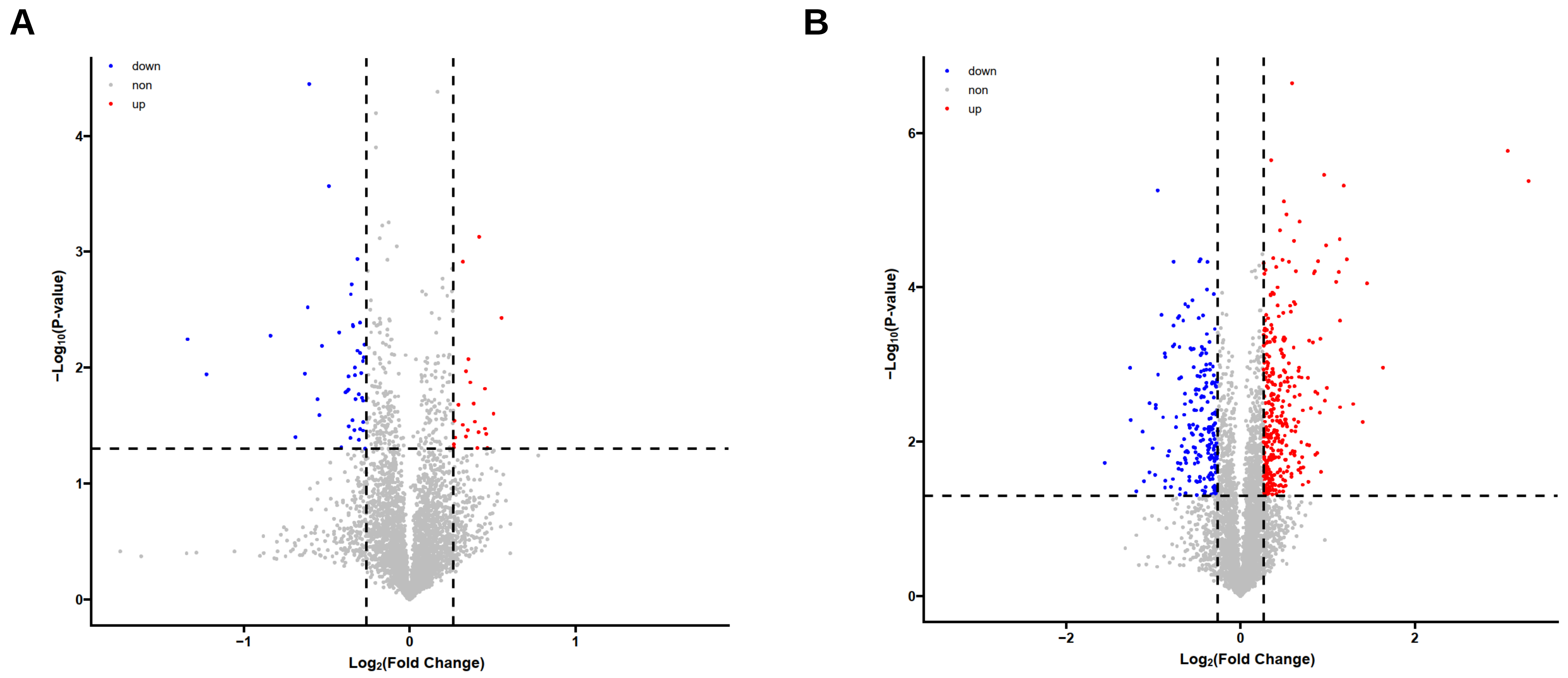


**Figure S6**. Volcano plots of the phosphopeptides distribution in *Glycine max* under sodium bicarbonate stress. (**A**) Leaf tissue; (**B**) Root tissue

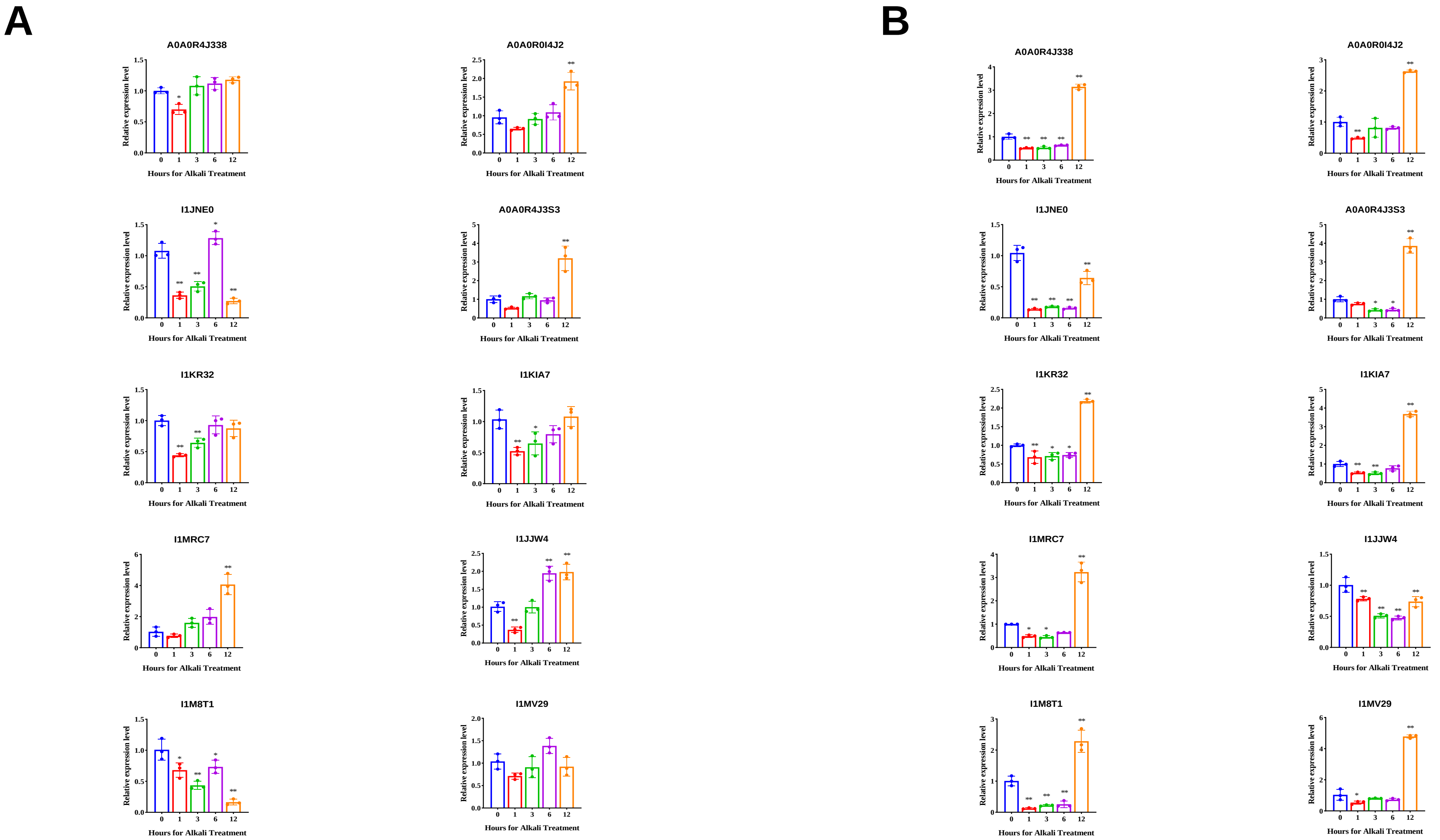


**Figure S7**. The RT-qPCR results showed the expression patterns of ten representative DEP encoding genes under sodium bicarbonate stress. (**A**) The expression patterns in the leaf tissue; (**B**) The expression patterns in the root tissue. GmGAPDH was used as an internal control. Three biological replicates and three technical replicates were performed for each experiment. Error bars show the Means ± SE of three biological replicates. * and ** indicate significant difference at 0.05 and 0.01 level by student's *t*-tests

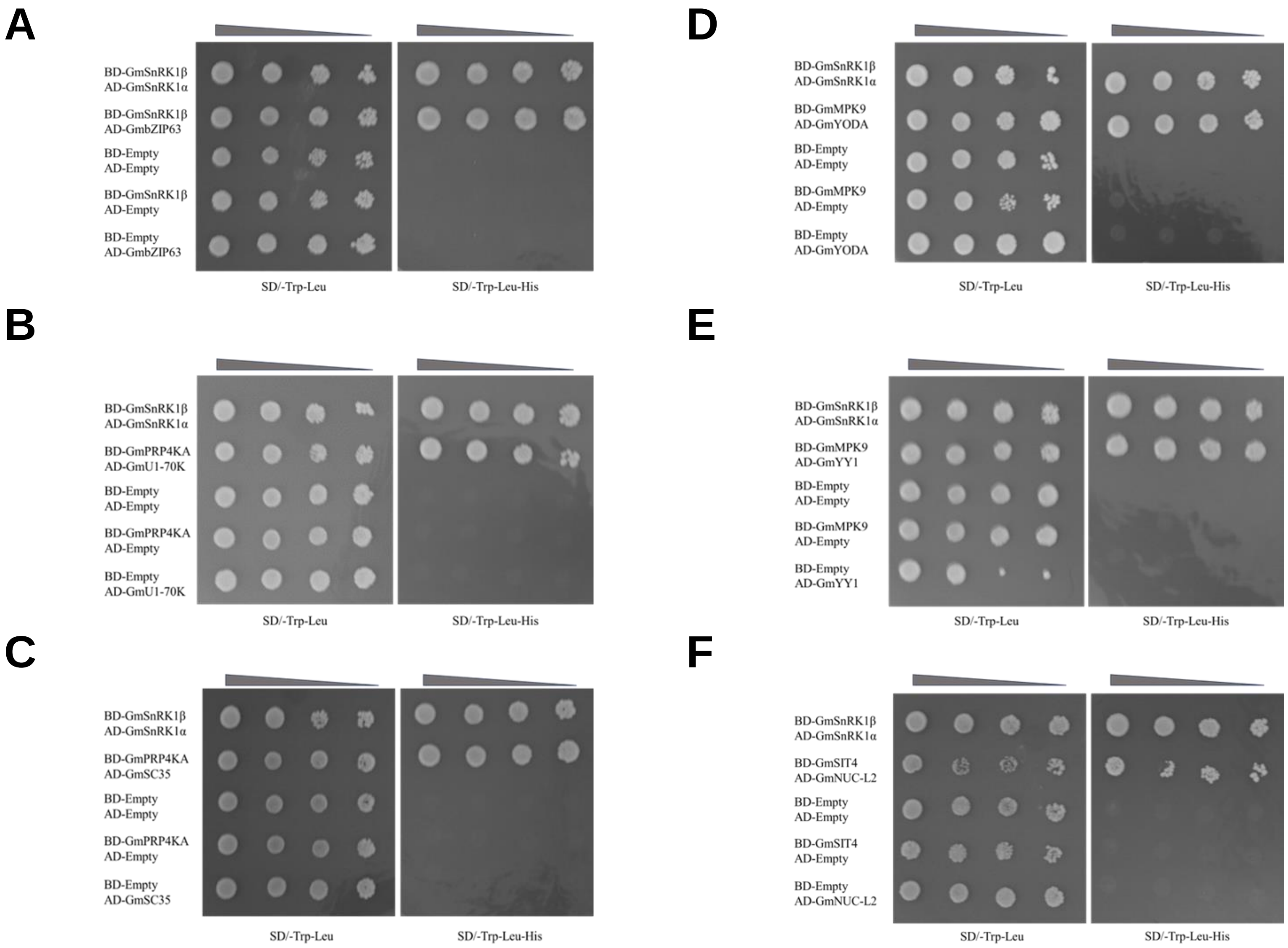


**Figure S8**. Physical interactions between K/Ps and their interacting proteins revealed by Y2H assay. The K/P-interacting protein combinations are as follows: (**A**) GmSnRK1β (A0A0R0L2D6) and GmbZIP63 (A0A0R0KPC1); (**B**) GmPRP4KA (A0A0R0I4J2) and GmU1-70K (I1LHR2); (**C**) GmPRP4KA (A0A0R0I4J2) and GmSC35 (I1KR32); (**D**) GmMPK9 (A0A0R0IHB9) and GmYODA (I1L345); (**E**) GmMPK9 (A0A0R0IHB9) and GmYY1 (C6THB4); (**F**) GmSIT4 (K7LBE9) and GmNUC-L2 (I1LPJ9)

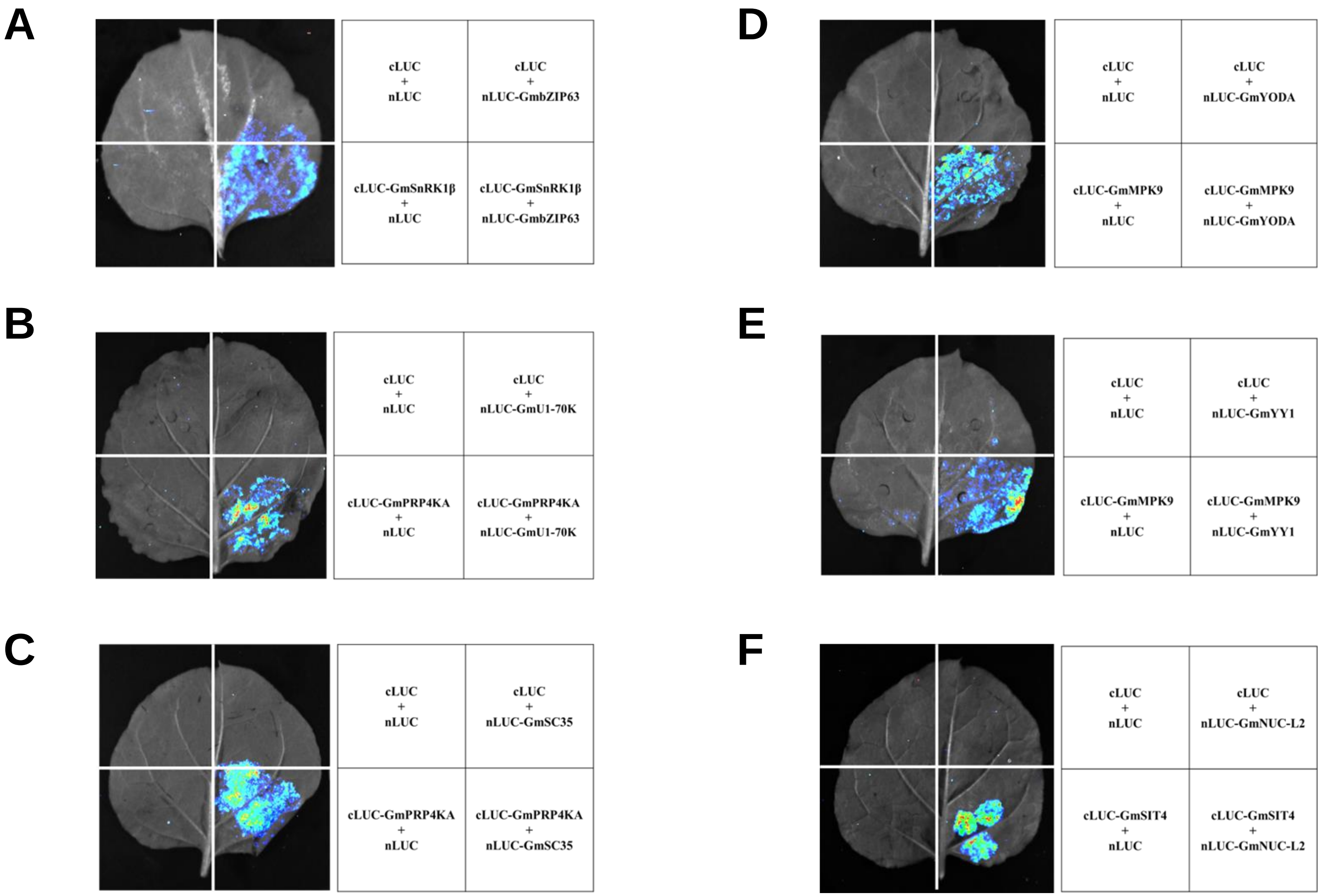


**Figure S9**. Physical interactions between K/Ps and their interacting proteins revealed by split-LUC complementation assay in tobacco leaves. The K/P-interacting protein combinations are as follows: (**A**) GmSnRK1β (A0A0R0L2D6) and GmbZIP63 (A0A0R0KPC1); (**B**) GmPRP4KA (A0A0R0I4J2) and GmU1-70K (I1LHR2); (**C**) GmPRP4KA (A0A0R0I4J2) and GmSC35 (I1KR32); (**D**) GmMPK9 (A0A0R0IHB9) and GmYODA (I1L345); (**E**) GmMPK9 (A0A0R0IHB9) and GmYY1 (C6THB4); (**F**) GmSIT4 (K7LBE9) and GmNUC-L2 (I1LPJ9)
